# Perspective: Lessons from COVID-19 of countries in the European region in light of findings from the health system response monitor

**DOI:** 10.3389/fpubh.2022.1058729

**Published:** 2023-01-06

**Authors:** Florian Tille, Ewout Van Ginneken, Juliane Winkelmann, Cristina Hernandez-Quevedo, Michelle Falkenbach, Anna Sagan, Marina Karanikolos, Jonathan Cylus

**Affiliations:** ^1^European Observatory on Health Systems and Policies, London School of Economics and Political Science, London, United Kingdom; ^2^European Observatory on Health Systems and Policies, Technische Universität Berlin, Berlin, Germany; ^3^Department of Healthcare Management, Technische Universität Berlin, Berlin, Germany; ^4^European Observatory on Health Systems and Policies, Brussels, Belgium; ^5^Department of Public and Ecosystem Health, Cornell University, Ithaca, NY, United States; ^6^European Observatory on Health Systems and Policies, London School of Hygiene and Tropical Medicine, London, United Kingdom

**Keywords:** health system, COVID-19, health system response monitor, resilience, policies, European Observatory on Health Systems and Policies

## Abstract

**Introduction:**

Decision-makers initially had limited data to inform their policy responses to the COVID-19 pandemic. The research community developed several online databases to track cases, deaths, and hospitalizations; however, a major deficiency was the lack of detailed information on how health systems were responding to the pandemic and how they would need to be transformed going forward.

**Approach:**

In an effort to fill this information gap, in March 2020, the European Observatory on Health Systems and Policies, the WHO European Regional Office and the European Commission created the COVID-19 Health System Response Monitor (HSRM) to collect and organise up-to-date information on how health systems, mainly in the WHO European Region, were responding to the COVID-19 pandemic.

**Findings:**

The HSRM analysis and broader Observatory work on COVID-19 shone light on a range of health system challenges and weaknesses and catalogued policy options countries put in place during the pandemic to address these. Countries prioritised policies on investing in public health, supporting the workforce, maintaining financial stability, and strengthening governance in their response to COVID-19.

**Outlook:**

COVID-19 is likely to continue to impact health systems for the foreseeable future; the ability to cope with this pressure, and other shocks, depends on having good information on what other countries have done so that health systems develop adequate policy options. In support of this, the country information on the COVID-19 HSRM will remain available as a repository to inform decision makers on options for actions and possible measures against COVID-19 and other public health emergencies. Building on its previous work on health systems resilience, the European Observatory on Health Systems and Policies will sustain its focus on analysing key issues related to the recovery from the pandemic and making health systems more resilient. This includes policy knowledge transfer between countries and systematic resilience testing, aiming at contributing to an improved understanding of health system response, recovery, and preparedness.

**Contribution to the literature in non-technical language:**

The COVID-19 Health System Response Monitor (HSRM) was the first database in the WHO European Region to collect and organise up-to-date information on how health systems were responding to the COVID-19 pandemic. The HSRM provides a repository of policies which can be used to inform decision makers in health and other policy domains on options for action and possible measures against COVID-19 and other public health emergencies. This initiative proved particularly valuable, especially during the early phases of the pandemic, when there was limited information for countries to draw on as they formulated their own policy response to the pandemic. Our perspectives paper highlights some key challenges within health systems that the HSRM was able to identify during the pandemic and considers policy options countries put in place in response. Our research contributes to literature on emergency responses and recovery, health systems performance assessment, particularly health system resilience, and showcases the Observatory experience on how to design such a data collection tool, as well as how to leverage its findings to support cross-country learning.

## Introduction

At the onset of the COVID-19 pandemic, decision-makers sought evidence to determine what, if anything, they could do to mitigate the pandemic's impact. The research and information community responded by quickly developing online databases to track cases, hospitalizations, and deaths, as well as to document a range of policies put in place by countries with respect to travel restrictions, fiscal measures, and lockdowns (1–4). Missing from these early initiatives, however, was information on what health systems were doing in response to the pandemic. This represented a crucial gap in knowledge.

Variations in health system responses may help to explain why some countries experienced relatively low hospitalisation and death rates even in the face of severe COVID-19 outbreaks, and why some countries largely avoided (at least some) COVID-19 waves. In addition, information on health system responses can contribute to wider understanding of why some countries have been better at maintaining the provision of essential health services and routine care, and how they avoided substantial service disruptions that resulted in increasing waiting times, which are already having substantial impact on health outcomes.

## Rationale, coverage and evolution of the health system response monitor

To fill this information gap and gain an understanding of effective policies that countries rolled out to mitigate the impact of the COVID-19 pandemic, the COVID-19 Health System Response Monitor (HSRM)^1^ was developed by the European Observatory on Health Systems and Policies, World Health Organisation Regional Office for Europe (WHO/Europe), and European Commission, to cover 50 countries' health systems and policy responses, primarily those in the WHO European Region (5). The HSRM was launched in March 2020 and remained operational and regularly updated well into 2022 (6). Although the focus was primarily on health system responses, the HSRM also captured wider public health initiatives on preventing transmission as well as relevant responses in other sectors, such as border controls, mobility and economy, amongst others (Table 1). It gathered evidence *via* publicly available information in English through a network of country experts from academia and WHO Country Offices. Observatory analysts worked with country experts to cheque and cross-reference, edit, and update posts for their respective countries. The country pages were then used to write a total of 70 concise comparative snapshots addressing specific policy questions covering a subset of countries in the database and aiming to distil concrete policy options.^2^ The content compiled in the HSRM platform was used extensively to inform a range of analytical outputs that compared COVID-19 responses across the monitored countries, including several Eurohealth editions (7, 8), a special issue in the journal Health Policy (9), a study on health system resilience (10), and a policy brief on backlogs and managing waiting lists during and beyond the COVID-19 pandemic (11).

**Table 1:** The HSRM topics and core information collected.

**Topics**	**Core information**
Preventing transmission	Key public health measures Measures in place to test and identify cases, trace contacts, and monitor the scale of the outbreak
Ensuring sufficient physical infrastructure and workforce capacity	Physical infrastructure Measures to address shortages Steps to maintain or enhance workforce capacity Workforce skill-mix and responsibilities Training and HR initiatives
Providing health services effectively	Planning and patient pathways for COVID-19 cases Maintaining essential services
Paying for services	How countries are paying for services Entitlements and coverage
Governance	Pandemic response plans Steering of the health system Emergency response mechanisms Regulation of health service provision to affected patients
Measures in other sectors	Borders Mobility (transport) Economy State aid Civil protection Cross-border collaboration

Other regional and global monitoring initiatives such as the pulse survey on the continuity of essential health services during the COVID-19 pandemic (12) and the ACT-Accelerator Global COVID-19 Access Tracker (GCAT) (13) provided similarly critical insights into the impact of the COVID-19 pandemic on health services and shed light on the challenges health systems were facing. The key difference between the three tools concerns their scope; while the HSRM has been organising information on the policies that countries chose in responding to the COVID-19 outbreak, the pulse survey assessed the impact of the pandemic on essential health services. The GCAT has been tracking progress towards the global targets for access to COVID-19 vaccines, treatments, tests and personal protective equipment (PPE).

## Findings from the HSRM on health policies put in place in response to the COVID-19 pandemic

The HSRM analysis and broader Observatory work on COVID-19 and on health system performance catalogued a range of policy measures taken in response to the pandemic and shone light on a number of long-standing challenges and weaknesses within health systems. These include, amongst others, issues related to the level of investment in public health, workforce capacity and flexibility, financial stability and equity, and governance constraints. In the following section, we highlight some of the key findings from these analyses in areas that countries prioritised in their response to COVID-19.

### Investing in public health

COVID-19 has exposed public health challenges and weaknesses on an unprecedented scale. The inability in many countries to slow disease transmission through test, trace, isolate mechanisms or to address the sharp increase in mortality in nursing homes during the pandemic can be seen as a reflection of the long-standing low priority given to public health, and long-term care in many European countries (14). The lack of investment in public health can also be seen in the poor state of the preparedness plans European countries had in place prior to the pandemic. Some of these plans ultimately could not be followed because they were either outdated, inadequate in terms of their level of detail (e.g., Italy, Spain), or otherwise were not suitable to address COVID-19 (Greece) (15).

Countries took a range of measures to improve their test, trace, isolate capabilities. To expand testing capacity, some countries, such as Germany, were able to take advantage of their extensive existing laboratory capacity at the onset of the pandemic, benefitting from its strong diagnostics industry. Similarly, many other countries repurposed existing laboratories (e.g., Croatia, France, Lithuania, Norway), while some smaller countries, at least initially, sent samples abroad (Ireland and Finland) (14). In Denmark, the national testing strategy gradually changed from a restrictive approach that included providing testing only to people with severe symptoms, to a much broader strategy offering testing also for people with mild symptoms in March 2020, asymptomatic individuals in April and others in May of that year (16).

Similarly, contact tracing had to be scaled up during the COVID-19 crisis. This was accomplished in different ways, including by diverting existing health workers, including administrative staff and those recently retired, to contact tracing; setting up *de-novo* structures (e.g., Serbia); contracting with outsourcing corporations (e.g., UK); or using existing capacity. In Germany, for example, the Federal Ministry of Health supported the local public health offices with €50 million to digitalize tracing operations and recruit additional tracers under an agreement between the federal and state governments. Similarly, in Austria the local health offices started performing contact tracing and monitor contacts in quarantine (14). Support for those who needed to isolate was nevertheless insufficient in many countries, resulting in some infected people continuing to engage in normal activities, particularly those on low-incomes or with precarious employment (14).

### Supporting the workforce

Many European health systems faced health workforce shortages prior to the COVID-19 pandemic (17). The pandemic exacerbated these existing shortages in many countries and regions due to a rising workload related to care for COVID-19 patients, the need to maintain essential health and social care services and to adopt new procedures, regulations, and hygienic standards but also because many health care workers were affected by COVID-19 either in their families or themselves, as in the early stages of the pandemic medical staff often worked without adequate protection (18). To scale-up and maintain the existing workforce capacity most countries used a variety of strategies to mobilise additional health workers. The most common approaches included: recruiting final year medical and nursing students, offering a transition from part-time to full-time work, modifying work schedules and cancelling leaves of absence, changing working patterns and bringing inactive or retired health professionals back to the workforce. In some countries the military and health professionals from the private sector helped to expand the available workforce capacity, and volunteers were recruited (e.g., Austria, Cyprus, Denmark, Estonia, Hungary, Montenegro) (19). Similarly, countries such as Italy, Romania and Spain re-deployed health workers to health facilities or regions with greater demand. Moreover, countries changed regulation to reskill and re-purpose health workers such as expanding the role of individual health professions and adapting or strengthening teamwork. England, Ireland and France, for instance, extended community pharmacists' scope-of-practise to renew certain prescriptions, while Germany shifted tasks from doctors to nurses to free up capacity (10, 18, 19). To protect health professionals from COVID-19 infections and mitigate further shortages, infection control policies and minimum standards of PPE use were defined and regular testing procedures were developed (20). Moreover, many countries such as France, Greece and Italy placed their health care workers high on the priority list for vaccine access or even mandated compulsory vaccinations for some or all health workers to promote uptake (21).

### Maintaining financial stability

The COVID-19 pandemic had major implications for economies and public finances, and in turn, for health system financial sustainability. Unemployment rose across Europe and wages declined, impacting the collection of social contributions and payroll taxes (22). Likewise, lockdowns and social distancing measures affected consumption behaviours and incomes, reducing taxes collected from these sources. As a result of these across-the-board effects on public revenues, to maintain health system financial sustainability, countries were required to borrow considerably and take on substantial public debt. This was made easier by temporary loosening of EU fiscal rules and extremely low borrowing costs, even in countries that had faced higher borrowing costs during the Great Recession (23).

**Figure 1:**
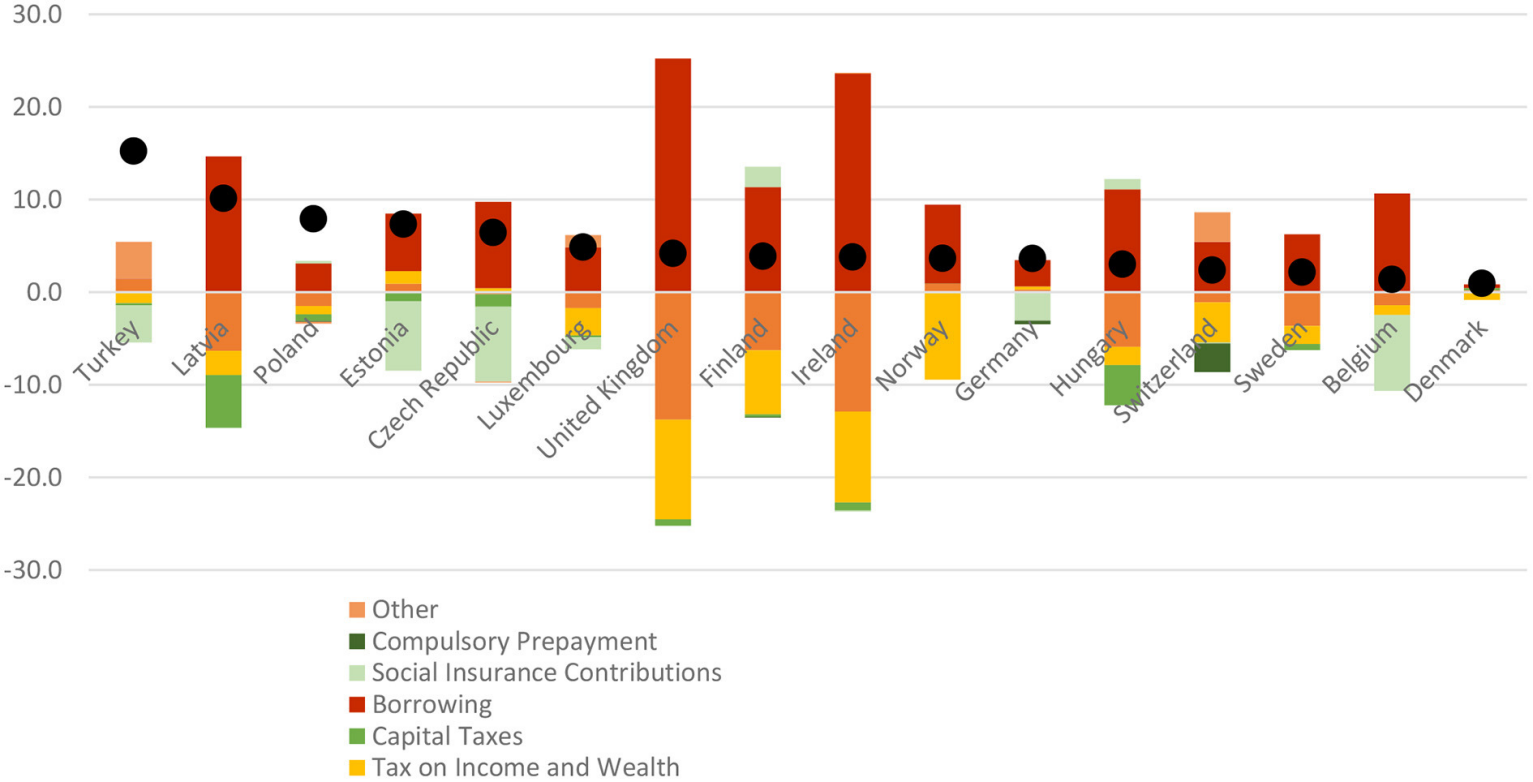
Changes to health revenues and per person public spending on health in OECD countries, 2019–2020. Author's analysis based on (24, 25). This figure does not include any reserves for health care spending which governments may have used as an additional source of financing.

To illustrate the extent of borrowing to finance health, Figure 1 shows changes in the mix of revenues used for health between 2019 and 2020 based on analysis of OECD data. Borrowing substituted for declines in social contributions and taxation most in the United Kingdom, Ireland, and Latvia, but even in countries with smaller shifts in the mix of health revenues, borrowing played an important role. Of note, in all OECD countries with data available, per person health spending by governments grew between 2019 and 2020.

### Strengthening governance with a focus on trust

There are many ways to strengthen governance. Specifically, during the pandemic, attention to open data sources, crisis and risk management, quality regulatory mechanisms, public sector management and communication and policy coherence, coordination and evaluation are essential. Aside from these aspects, one of the key but often overlooked areas, for strengthening effective governance during the pandemic has been to solidify public trust in decision-making authorities (26), requiring close scrutiny. For COVID-19, the HSRM analysis found that an increase in trust in government and health officials, in countries like Denmark, Switzerland or Italy, led to a greater acceptance of government-mandated measures (e.g., regulations on testing, lockdowns, and vaccination) and less politization of the pandemic and its societal impacts (27, 28). In turn, these countries generally experienced better outcomes, including higher vaccination rates, and as a result, lower hospitalisation, and mortality rates (28).

Some positive changes in countries to increase trust could be observed through the HSRM contents. For example, providing open access to data and displaying how the data is used in response measures to COVID-19 was effective at improving transparency of decision-making, which was shown especially by Scandinavian countries Likewise, the dissemination of credible and consistent scientific advice by key government actors was important in Germany where a well-known virologist was seen as a widely trusted source of information on COVID-19. Transparent and effective public communication was also crucial in response to disinformation (29). Examples of this can again be found in Germany and Switzerland. Finally, policy evaluation played an important role so that citizens could be reassured that policy decisions were based on available evidence and working towards delivering the desired outcomes (30); the Danish Strategy for managing COVID-19 is prominent example of this (31).

## Brief outlook on the future role of HSRM for health system recovery and preparedness and the Observatory's work on resilience

COVID-19 is likely to continue to impact health systems for the foreseeable future; their ability to cope with increases in demands for services, and to prevent, prepare for and respond to other shocks, depends on having good information on what other countries have done so that health systems may develop judicious policies. In support of this, the country information on the COVID-19 HSRM will remain available as an archive of policy responses and there will also be a focus on ongoing analysis of key issues related to the recovery from the pandemic and improving health systems resilience.

### Wider Observatory activities on health systems resilience

While COVID-19 has brought the topic of health systems resilience to the forefront of many organizations' analytical priorities, the Observatory's work on resilience started well before the pandemic, reaching back to the publication of the first edition of the State of Health in the EU (SoHEU) country profiles with the OECD and European Commission in 2017 (32). There, as well as in the subsequent second edition of the profiles (33), the analysis of resilience focused on the most pressing challenges specific to each country, as well as on more general pressures such as population ageing. The analysis for each country in the SoHEU series explored the long-term stability of health system resources, the ability to operate efficiently, and governance issues. Largely informed by the content compiled in the HSRM platform and its various analytical outputs in 2021, the third edition of the profiles looked at health systems resilience during the COVID-19 pandemic and focused mainly on countries' preparedness and management responses to the pandemic, presenting policy measures and strategies that were implemented within the health system to contain the pandemic and respond to the health care needs of COVID-19 and other patients (34).

In addition, in early 2020, the Observatory policy brief “Strengthening health systems resilience: key concepts and strategies” sought to dispel some of the confusion around the concept of health systems resilience and to identify a list of key resilience strengthening strategies based on the lessons from previous shocks (35). In combination with the core HSRM material and its derivative outputs described above, this conceptual work continues to inform the Observatory's study of health systems resilience to COVID-19. More recent work refined the original, generic list of strategies into one pertaining specifically to the COVID-19 pandemic (10). By considering resilience through the lens of the core health system functions (governance, financing, resource generation and service delivery) the strategies endeavoured to unpack the complexity of responses and pinpoint entry points for improvements and reforms. Indeed, while the focus of this analysis was on policy responses during the crisis, the study also seeks to draw lessons going forward, appreciating the pandemic as an opportunity for health system strengthening.

### Introducing the Observatory's work on systematic health system resilience testing

Looking ahead, systematic resilience testing should be considered as a useful tool to identify health system weaknesses before the next major health system shock. To this end, using the newly developed Health System Performance Assessment Framework for Universal Health Coverage (36) as a basis, the Observatory is developing a methodological approach to resilience testing that helps policy makes to identify health system weaknesses in light of specific health system shocks or challenges including recessions and cost-of-living crises, antimicrobial resistance (AMR), climate change, pandemics, and others. The project is funded by the European Commission and carried out jointly with the OECD. Beginning in 2023, the project aims to offer EU countries a systematic and harmonised approach that they can use to better understand the performance of their health systems in the face of health system shocks. Future work on health system resilience may also focus on health emergencies preparedness and other topics within health systems, such as the Primary Health Care Monitoring Framework and Indicators (PHCMFI) (37), International Health Regulations Monitoring and Evaluation Framework (IHR MEF) (38), and Health Emergency and Disaster Risk Management Framework (EDRM) (39).

The Observatory will continue to study health system resilience both retrospectively (i.e., how well have countries responded to COVID-19?), as well as prospectively (how can health systems better prepare for future shocks?) to contribute to an improved understanding of health system response, recovery, and preparedness in the European region (40).

## Data availability statement

The datasets presented in this study can be found in online repositories. The names of the repository/repositories and accession number(s) can be found at: https://eurohealthobservatory.who.int/monitors/hsrm/hsrm-countries.

## Author contributions

FT and JC developed the concept and outline of the article. FT, EG, JW, CH-Q, MF, AS, MK, and JC drafted sections of the manuscript, with FT and JC finalising the article. All authors read and approved the final manuscript.

## Footnotes

^1^ https://eurohealthobservatory.who.int/monitors/hsrm/overview

^2^ https://eurohealthobservatory.who.int/monitors/hsrm/analyses
